# A test of balanced fitness limitations theory: Pollen limitation in plants

**DOI:** 10.1002/ece3.10911

**Published:** 2024-01-31

**Authors:** Jay A. Rosenheim, Neal M. Williams, Joshua M. Rapp, Sebastian J. Schreiber

**Affiliations:** ^1^ Department of Entomology and Nematology University of California Davis Davis California USA; ^2^ Department of Evolution and Ecology University of California Davis Davis California USA; ^3^ Present address: Mass Audobon Lincoln Massachusetts USA

**Keywords:** balanced limitations theory, essential resources, pollen limitation, pollen supplementation, pre‐pollination costs, self‐compatibility

## Abstract

When reproductive success is determined by the relative availabilities of a series of essential, non‐substitutable resources, the theory of balanced fitness limitations predicts that the cost of harvesting a particular resource shapes the likelihood that a shortfall of that resource will constrain realized fitness. Plant reproduction through female function offers a special opportunity to test this theory; essential resources in this context include, first, the pollen received from pollinators or abiotic vectors that is used to fertilize ovules, and, second, the resources needed to provision the developing seeds and fruit. For many plants realized reproductive success through female function can be readily quantified in the field, and one key potential constraint on fitness, pollen limitation, can be assessed experimentally by manually supplementing pollen receipt. We assembled a comparative dataset of pollen limitation using only studies that supplement pollen to all flowers produced over the plant's reproductive lifespan. Pre‐ and post‐pollination costs were estimated using the weight of flowers and fruits and estimates of fruit set. Consistent with expectations, we find self‐incompatible plants make greater pre‐pollination investments and experience greater pollen limitation. However, contrary to theoretical expectations, when variation due to self‐compatibility is accounted for by including self‐compatibility in the statistical model as a covariate, we find no support for the prediction that plants that invest more heavily in pre‐pollination costs are subject to greater pollen limitation. Strong within‐species, between‐population variation in the expression of pollen limitation makes the quantification of mean pollen limitation difficult. We urge plant ecologists to conduct more studies of pollen limitation using whole‐plant pollen supplementation to produce a richer comparative dataset that would support a more robust test of the balanced limitations hypothesis.

## Graphical abstract

We assembled a comparative dataset of pollen limitation in plants to test a central prediction from the theory of balanced fitness limitations, namely that the cost of harvesting a particular resource shapes the likelihood that a shortfall of that resource will constrain realized fitness. We find no support for the prediction that plants that invest more heavily in pre‐pollination costs are subject to greater pollen limitation.

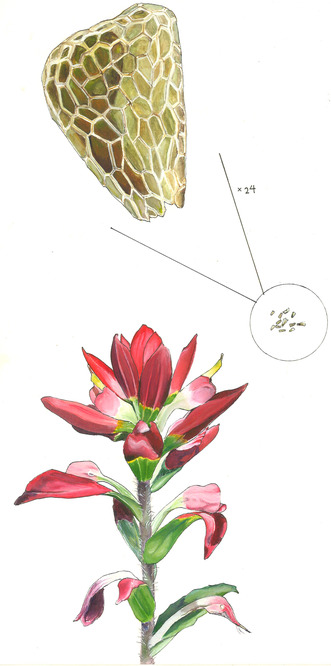

## 1 INTRODUCTION

The evolutionary ecology literature is filled with controversies regarding which factors emerge as consistent limits to the reproductive success of different organisms. In cases where ecologists have considered the role of essential, non‐substitutable resources needed for successful reproduction in an unpredictably varying environment, these controversies have historically often pitted different camps, each championing a preeminent role for a particular limiting factor, against each other (e.g., Chirichella et al., 2023; Fay et al., 2015; Harpole et al., 2011; Kirkwood, 2008; Rosenheim, 1996; Sevenster et al., 1998; Thomas et al., 2022; Wehmann et al., 2022). Against this backdrop of debates, theoretical work has coalesced on the prediction that natural selection will favor traits that balance multiple limiting factors, such that different factors limit fitness at different times or places (Dawkins, 1995; Haig & Westoby, 1988). But not all factors are predicted to have equal likelihoods of limiting fitness; rather, balanced limitations theory predicts that it is the physiological cost of alleviating the impact of a particular limiting factor that shapes the likelihood of that factor emerging as the limit to fitness (Ellers et al., 2000; Rosenheim, 2011; Rosenheim et al., 2010; Segoli & Wajnberg, 2020). The more expensive it is to harvest a particular resource, the more likely a shortfall of that resource is predicted to limit reproductive success. Similar predictions have been obtained in the fields of engineering and economics (Elishakoff, 2004; Teunter et al., 2010), suggesting that a positive relationship between cost and limitation is fundamental to optimization under uncertainty.

Tests of this central prediction of the balanced limitations hypothesis have, however, proven to be difficult to conduct in nature. One exception has come from a new study of egg limitation in insect parasitoid wasps: Segoli et al. (in press) demonstrated that egg costs (measured as the size of an egg relative to the size of the female parasitoid) are positively correlated with the likelihood that a female parasitoid's lifetime reproductive success would be limited by the female's finite supply of eggs. In most cases, however, measuring realized lifetime reproductive success in nature is difficult, and determining what factor limits reproductive success for a given individual or across a population is even more difficult.

Here we present a comparative test of the hypothesized positive relationship between cost and limitation that capitalizes on the tractability of measuring reproductive success of plants through female function, along with the impact of a key constraint on that reproduction: pollen limitation. Plant ecologists have long debated the role of pollen limitation as a constraint on female reproductive success. Pollen limitation occurs when lifetime seed production by a plant is constrained by inadequate pollen receipt, as opposed to limitation by the resources needed to provision seeds and fruits. Like many other limiting factor debates, different authors have advocated essentially all possible viewpoints, including that pollen limitation should be completely absent from plant populations (Janzen, 1977; Willson, 1979), should be ubiquitous (Burd, 2008), or should be a 50% risk experienced by all individuals (Thomson, 2001). Pollen limitation can be quantified experimentally by manually supplementing pollen received by some plants and comparing their lifetime seed output with other, comparable members of the same plant population that did not receive supplemental pollen (open pollination controls). The balanced limitations theory makes two predictions. First, plant populations are predicted to evolve life history traits that allow them to balance the impact of pollen limitation versus fitness limitation by the finite supply of resources used to provision seeds and fruits. Thus, we do not expect pollen limitation to be completely absent from plant populations (0% of plants pollen limited), nor do we expect it to be universal (100% of plants pollen limited). Instead, theory predicts intermediate mean levels of pollen limitation in all plant populations. Second, pollen limitation is predicted to be more common for those plants where the physiological costs of securing pollen (pre‐pollination costs of seed production, including the costs of producing attractive flowers and rewards for pollinators) are large, relative to the costs of provisioning seeds and fruits and building protective or dispersal structures for seeds (post‐pollination costs of seed production; Rosenheim et al., 2014, Schreiber et al., 2015). Thus, pollen limitation is predicted to be relatively rare for plants that whose flowers are small and inexpensive relative to the costs of the larger seeds and fruits (e.g., wild *Prunus* spp.), whereas pollen limitation is predicted to be much more common for plants whose flowers are large and expensive relative to the costs of smaller seeds and fruits (e.g., many orchid species).

The empirical literature on pollen limitation in flowering plants is vast (e.g., Bennett et al., 2020); however, because the theory we wish to test is explicitly concerned with limits to lifetime reproductive success, pollen limitation must be measured using an exacting protocol: experimental supplementation of pollen must be performed across all (or nearly all) flowers produced by a plant over its entire reproductive life. Supplementing pollen to a small subset of flowers (e.g., individual flowers, or all flowers produced during a single year by a polycarpic perennial plant) has been shown to grossly overestimate pollen limitation, as plants often allocate extra resources to supplemented flowers at the expense of other flowers produced at different locations or times (Knight et al., 2006; Webber et al., 2020). Because few researchers have supplemented pollen across all flowers produced by a plant, and because pre‐pollination and post‐pollination costs are rarely reported, the dataset we were able to build was relatively small. Perhaps in part as a consequence, the results we present here are largely null. Our goals in presenting these results are two‐fold: first, to avoid the distorting effects of withholding non‐significant results from the published scientific literature, and second, to encourage plant biologists to measure pollen limitation using pollen supplementation across all flowers.

## 2 MATERIALS AND METHODS

### 2.1 Literature survey of pollen limitation

We used the GloPL pollen limitation database described by Bennett et al. (2018) to locate published records of pollen limitation estimated using whole‐plant pollen supplementation for monocarpic plant populations (either annuals or monocarpic perennials). A literature search using the Web of Science search engine (search terms: “pollen limit*” OR “pollen supplem*” OR “supplem* poll*” OR “hand poll*”) extended the literature coverage from when GloPL's review ended (2017) through February 26, 2021. We followed GloPL's approach of excluding crop plants. We also obtained unpublished estimates of pollen limitation from some researchers. Whenever possible, we used the total number of seeds produced per plant as our response variable for measuring reproductive success; when this was not available, we chose what we deemed to be the closest metric (e.g., seeds per fruit in studies that reported that mean fruit number was equal across the pollen supplementation and open pollination treatments). We computed pollen limitation as [(reproduction with pollen supplementation) − (reproduction under open pollination)]/(reproduction under open pollination). This metric of pollen limitation differs slightly from the metric introduced by Larson and Barrett (2000); we use it here because it is the metric used in the models that generated the predictions that we test here (Rosenheim et al., 2014; Schreiber et al., 2015). Positive values of this metric signify that seed production under open pollination was constrained by pollen receipt; a value of 1.0 indicates that seed production was increased by 100% (i.e., seed production was doubled) by the pollen supplementation treatment. The magnitude of this pollen limitation metric is expected to closely track the proportion of individuals in the plant population that are pollen limited (Rosenheim et al., 2014; whether an individual plant is pollen limited or not is difficult to assess experimentally, because a single plant can only be observed in one condition—open pollinated, or pollen supplemented—but not both). Pollen limitation is expected to vary strongly across time and space. Some studies included estimates of pollen limitation for multiple populations of the same species; we included these replicate estimates when they were made during different years or in populations separated by at least 1 km. Data were extracted from published tables and from figures (using WebPlotDigitizer v4.6 (Rohatgi, 2021)), and in many cases authors shared original data files, allowing us to compute more accurate estimates. In some studies authors measured a plant size covariate to reduce variation in total seeds produced per plant due to factors other than pollen receipt; we used covariates in linear statistical models of seed production in computing pollen limitation only if the covariate was a measure of a vegetative structure (e.g., total plant dry weight, total number of leaves per plant, plant diameter) and not any trait directly related to the plant's strategy for pollen harvest (e.g., flower number). All data extraction was performed twice, by two of the authors (JAR and NMW) working independently, and any discrepancies in results resolved.

### 2.2 Cost estimates

Costs of seed production were estimated using the dry weight of the flower (pre‐pollination costs) and the mature fruit (post‐pollination costs). Dry weight is the only measure of cost that is commonly reported in the literature; shortcomings of this metric have been discussed (Burd, 2016; Rosenheim et al., 2014). For flowers that have both male and female reproductive structures present, pre‐pollination costs of female reproduction were generally estimated as half of the total dry weight of the flower or, in some cases, as the weights of specifically female structures (i.e., the pistil) plus half of the weight of the calyx and corolla. For species that retain floral structures on the mature fruit, we subtracted flower weight from fruit weight when calculating post‐pollination costs (to avoid double‐counting). To calculate the proportion of the total cost of seed production that occurs pre‐pollination we should also incorporate the influence of fruit set (proportion of costs occurring pre‐pollination = (dry weight of female parts of flower)/((dry weight of female parts of flower) + (fruit set) (fruit dry weight)); see Haig & Westoby, 1991). Fruit set estimates were often reported in the study reporting pollen limitation. When flower and fruit dry weights or fruit set estimates were not reported in the paper that reported pollen limitation, we searched the literature for the needed information and then contacted the authors for unpublished data if published data were not available. Several authors returned to their field sites to gather additional data for us (see Acknowledgments). We supplemented data obtained from authors by conducting our own field studies to obtain needed estimates. For pollen limitation studies originally carried out in California, we returned to the same plant populations used to obtain the original pollen limitation estimates.

Because including fruit set as part of our pre‐pollination cost calculations risks injecting some circularity into our test (low fruit set elevates the pre‐pollination cost metric and can clearly be associated with shortfalls of pollen receipt), we conducted supplementary analyses in which fruit set was excluded from the cost calculation (i.e., fruit set was assumed to be 100%).

### 2.3 Data analysis

**FIGURE 1:**
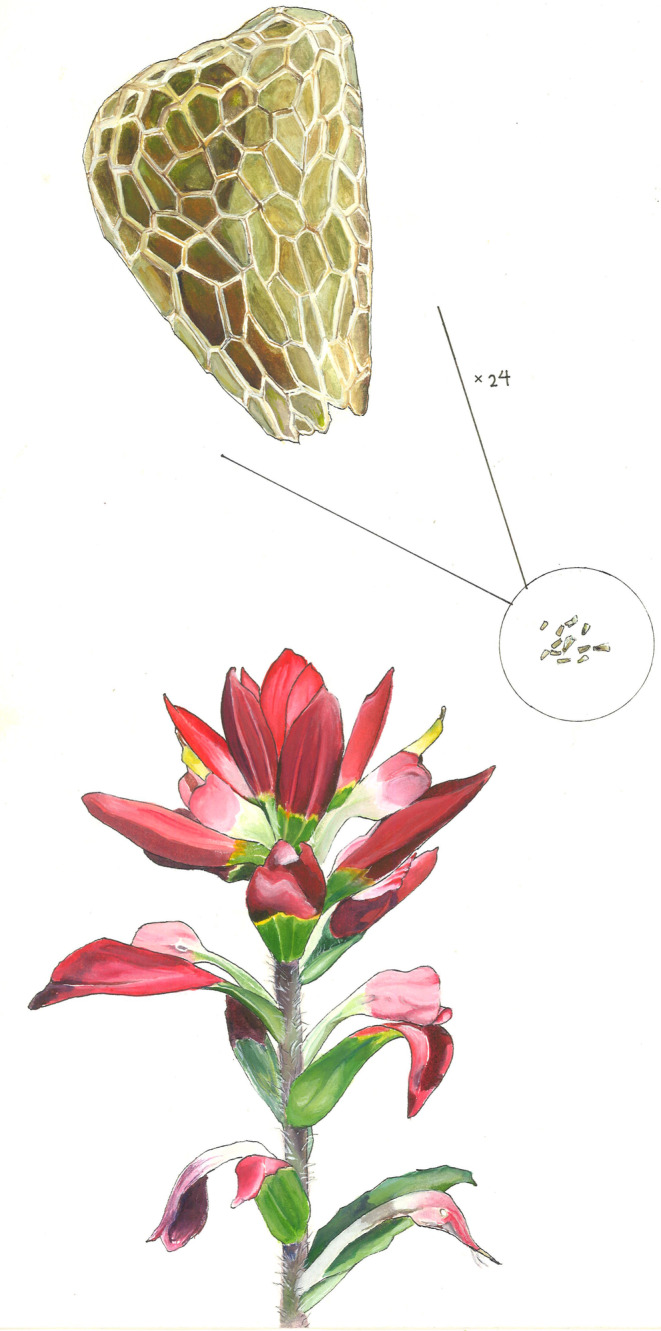
Example of a plant, *Castilleja indivisa*, that was included in our comparative analysis. This species has the largest value in the comparative dataset for the proportion of the total cost of seed production that occurs pre‐pollination (0.682); it has large attractive structures (below) and tiny seeds (×24 magnified view shown above) and very high levels of observed pollen limitation (1.69; Adler, 2000). Original artwork by Leah Y. Rosenheim.

Phylogenetic relationships among plant taxa were estimated using the mega‐tree GBOTB.extended.tre as implemented in the R package “*V.PhyloMaker*,” with baseline options (scenario 1 and nodes.info.1; Jin & Qian, 2019). Taxonomic names of plants for which we obtained pollen limitation estimates were updated to be compatible with GBOTB.extended.tre using The World Flora Online (http://www.worldfloraonline.org/; Accessed 16 March 2023). Phylogenetic linear mixed models were fit using R package “*phyr*” (Ives et al., 2022). The model included pre‐pollination costs as the primary predictor; self‐compatibility (self‐compatible, self‐incompatible, or unknown) as a covariate; and species ID as a random effect to accommodate the multiple pollen limitation estimates obtained for some species. Self‐incompatible plant populations have been found consistently to express higher levels of pollen limitation (Burd, 1994; Burns et al., 2019; Knight et al., 2005; Larson & Barrett, 2000). Plant populations that were capable of fully autonomous self‐pollination were excluded from our analysis; thus, the self‐compatible species studied here are still dependent on pollen vectors for fertilization of ovules. Our response variable was pollen limitation, as described above [(reproduction with pollen supplementation) − (reproduction under open pollination)]/(reproduction under open pollination). An alternative response variable, the Log Response Ratio (LRR = ln(reproductive output of pollen supplemented plants) − ln(reproductive output of open pollinated plants)) that is widely employed in metanalyses (Koricheva & Gurevitch, 2014) produced qualitatively identical results (data not shown). We built a complementary model to ask if self‐compatible plant species were associated with smaller pre‐pollination costs. We also computed linear mixed models ignoring plant phylogeny as points of comparison. Means are reported ±1 SE throughout. The full comparative dataset, including pollen limitation and pre‐pollination cost estimates for 41 populations (18 species; e.g., Figure 1) is presented in Table S1.

## 3 RESULTS

**FIGURE 2:**
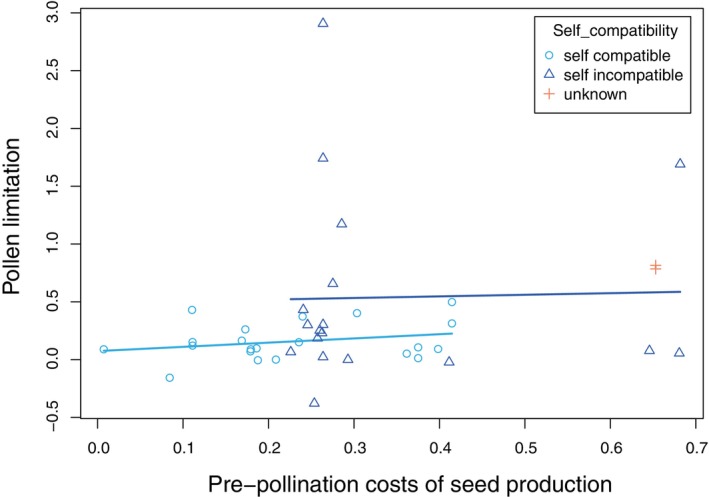
Relationship between pre‐pollination costs of seed production and field‐assessed impact of shortfalls of pollen receipt (pollen limitation) on lifetime seed production by self‐compatible and self‐incompatible plants.

A phylogenetic linear mixed model including the effects of variable fruit set on pre‐pollination costs revealed no support for the hypothesis that pre‐pollination costs shape the realized incidence of pollen limitation in nature (effect for pre‐pollination costs = 0.316 ± 0.709, *z* = 0.45, 1‐tailed *p* = .33, Figure 2, Tables S2–S5). Very similar results were obtained in an analysis that excluded the effects of variable fruit set on pre‐pollination costs and in analyses that fit non‐phylogenetic linear mixed models (Tables S2–S5). The weak positive trend observed across all plant populations (Figure 2) appears to be explained largely by the self‐incompatible populations having both greater pre‐pollination costs than do self‐compatible populations (self‐incompatible, mean = 0.337 ± 0.037, *n* = 18; self‐compatible, mean = 0.241 ± 0.030, *n* = 21; main effect for self‐incompatibility = 0.181 ± 0.022, *z* = 8.30, 1‐tailed *p* < .0001) and greater observed levels of pollen limitation than do self‐compatible populations (self‐incompatible, mean = 0.538 ± 0.195, *n* = 18; self‐compatible, mean = 0.157 ± 0.036, *n* = 21; main effect for self‐incompatibility = 0.339 ± 0.211, *z* = 1.60, 1‐tailed *p* = .05; Tables S2–S5). Thus, self‐incompatible plants pay larger costs to secure fertilizations but still experience greater shortfalls of pollen receipt than do self‐compatible plants. Nevertheless, neither self‐compatible nor self‐incompatible species showed clear positive relationships between pre‐pollination costs and the magnitude of pollen limitation.

## 4 DISCUSSION

Our comparative analysis provides no support for the predicted positive relationship between pre‐pollination allocations and the incidence of pollen limitation. Although our dataset was small, it was still large enough to confirm two well‐supported results in plant reproductive ecology, namely that self‐incompatible plants often make larger investments in floral displays to secure adequate fertilization of ovules (Goodwillie et al., 2010) and that, despite these larger investments, self‐incompatible species often face elevated loss of reproduction due to shortfalls of pollen receipt (Burd, 1994; Burns et al., 2019; Knight et al., 2005; Larson & Barrett, 2000).

It is unlikely that the plants included in our comparative analysis are a random set of plant species with respect to the impact of pollen limitation; plant ecologists interested in pollen limitation presumably tend to choose study subjects that they think will exhibit the phenomenon they wish to study. Nevertheless, mean levels of pollen limitation in our dataset were low for self‐compatible populations (0.157 ± 0.036, *n* = 21) and moderate for self‐incompatible populations (0.538 ± 0.195, *n* = 18). These mean values are much lower—indeed, approximately an order of magnitude lower—than those reported in much larger surveys that included studies that supplemented pollen to small subsets of the flowers produced by a plant over its reproductive life (i.e., single flowers, single inflorescences, or single years for polycarpic species; e.g., Bennett et al., 2020). Thus, although all plant species should be expected to have some, non‐zero risk of experiencing pollen limitation, this risk appears in most species to be modest in magnitude. There are, however, some striking exceptions, where plants express severe pollen limitation. Understanding the causes of these instances of severe pollen limitation is an important research goal.

Why do we find no support for the prediction of a positive relationship between pre‐pollination costs and pollen limitation? There are at least five non‐mutually exclusive possibilities. First, there may be fundamental errors in the assumptions underlying the life history theory that generates this prediction. For example, predictions of pollen limitation emerge from models that assume strict non‐substitutability of essential resources and that do not consider the role of phenotypic plasticity in coping with uncertainty in pollen availability. Plastic responses such as delayed selfing, prolongation of floral lifespan, production of fewer but larger seeds, and, for polycarpic species, reallocation of resources between years, could allow plants to moderate the fitness costs of shortfalls of pollen receipt (Goodwillie & Weber, 2018; Torres‐Díaz et al., 2011). Models incorporating these and other potentially important factors might change predicted evolutionary optima. Second, the studied plant populations may not be at an evolutionary optimum with respect to the trade‐off between pre‐pollination and post‐pollination allocations (Knight et al., 2005). Many of the studied plant populations occupied human‐disturbed habitats, and other plant populations were studied outside their native range (Data S1). Furthermore, competing trade‐offs that are not considered here, including those connected to male reproductive success, may also change allocations to attraction of pollen vectors. Selection to export pollen efficiently to fertilize ovules on other plants may be just as important as selection to import sufficient pollen to fertilize the plant's own ovules (Delph & Ashman, 2006; Rodríguez‐Otero et al., 2023). Third, the previously discussed problems with measuring the costs of pre‐ versus post‐pollination investments in female reproduction almost certainly inject meaningful errors into our measurements (Burd, 2016; Rosenheim et al., 2014). Fourth, plant species with high pre‐pollination costs (>0.5) are relatively rare (only 3 of the 17 plant species studied here), making it more difficult to resolve the role of those costs. And fifth, strong spatial and temporal variation in the expression of pollen limitation may make it more difficult to resolve underlying patterns, especially with a small dataset like this one.

Although we cannot authoritatively assess the relative roles of these five factors, our dataset does underscore the importance of variation in the expression of pollen limitation for a given species across populations. Our dataset includes four plant species for which we have pollen limitation estimates for at least four populations: *Leptosiphon bicolor* (pollen limitation estimates = −0.006, 0.071, 0.089, and 0.097; *n* = 4), *Leptosiphon jepsonii* (0.013, 0.051, 0.091, 0.105, 0.313, and 0.498; *n* = 6), *Leptosiphon parviflorus* (0.066, 0.185, 0.230, 0.252, 0.300, 0.431, 1.173; *n* = 7), and *Ipomopsis aggregata* (−0.379, 0.023, 0.302, 0.656, 1.741, 2.908; *n* = 6 populations). Three of these four species (all but *L. bicolor*) exhibit moderate to strong variation in pollen limitation; indeed, *I. aggregata* exhibits both the very lowest (−0.379) and the very highest pollen limitation value (2.908) in the entire dataset. As expected, unpredictable variation in the environment generates major variation in the expression of pollen limitation. Thus, although plant populations may represent some of the most tractable opportunities for testing predictions from balanced fitness limitations theory, even here sustained effort will be required to build a robust dataset.

We hope this paper will motivate researchers to conduct the field studies that will, collectively, create a richer dataset that will support a stronger test of balanced fitness limitations theory. We propose that the plant species that will be most valuable in strengthening the comparative dataset will be those that (a) are self‐incompatible; (b) have high pre‐pollination costs (expensive flowers) and modest post‐pollination costs (inexpensive seeds and fruits); and (c) are experimentally tractable (monocarpic, with a total number of flowers produced that is sufficiently modest that pollen can be supplemented to all flowers produced over the plant's reproductive life). Plants with this combination of traits are not common but are not truly rare either (Rosenheim et al., 2014). Supplementation of pollen at the level of the whole plant and encompassing the full period of a plant's reproductive effort will be critical to establishing estimates of the impact of pollen limitation on lifetime reproductive success that are not distorted by reallocation of plant resources within individual plants (Knight et al., 2006).

## AUTHOR CONTRIBUTIONS

**Jay A. Rosenheim:** Conceptualization (equal); data curation (equal); formal analysis (lead); funding acquisition (supporting); project administration (supporting); supervision (supporting); writing – original draft (lead); writing – review and editing (lead). **Neal M. Williams:** Conceptualization (equal); data curation (equal); funding acquisition (supporting); project administration (equal); supervision (equal); writing – review and editing (supporting). **Joshua M. Rapp:** Conceptualization (equal); data curation (equal); formal analysis (supporting). **Sebastian J. Schreiber:** Conceptualization (equal); funding acquisition (lead); project administration (lead); supervision (supporting); writing – review and editing (supporting).

## CONFLICT OF INTEREST STATEMENT

The authors declare no conflict of interest.

## Supporting information


Data S1:

Tables S1–S5:

## Data Availability

All data files are included in the supplementary material files included with the submission.
