# Supplementary material for: A test of balanced fitness limitations theory: Pollen limitation in plants
Source: Ecol Evol. 2024 Jan 31;14(2):e10911. doi: 10.1002/ece3.10911 (PMC10830348; doi:10.1002/ece3.10911)
Supplement: Supplementary file 2 — Tables S1–S5: [file ECE3-14-e10911-s002.zip › ece310911-sup-0003-TablesS2-S5.docx]

**S2 Table. Phylogenetic linear mixed model examining the influence of pre-pollination costs of seed production, including the effects of fruit set, on pollen limitation observed in nature.** The baseline condition is for plants to be self-compatible.

> PL1 <- pglmm(PL ~ PrePollinationCostsInclFruitSet + Self.compatibility + (1|GenusSpecies__), data = PollenLim, family = "gaussian", cov_ranef = list(GenusSpecies = tree.a$scenario.1), REML = TRUE, verbose = TRUE, s2.init = .1)

> summary(PL1)

Linear mixed model fit by restricted maximum likelihood

Call:PL ~ PrePollinationCostsInclFruitSet + Self.compatibility

logLik AIC BIC

-31.96 77.92 81.90

Random effects:

Variance Std.Dev

1|GenusSpecies 0.007901 0.08889

residual 0.316043 0.56218

Fixed effects:

Value Std.Error Zscore Pvalue

(Intercept) 0.080728 0.218945 0.3687 0.7123

Pre-pollination costs 0.316269 0.708923 0.4461 0.6555

Breeding system:

Self-incompatibility 0.338595 0.211086 1.6041 0.1087

Self compatibility unknown 0.550283 0.530911 1.0365 0.3000

**S3 Table. Phylogenetic linear mixed model examining the influence of pre-pollination costs of seed production, excluding the effects of fruit set, on pollen limitation observed in nature.** The baseline condition is for plants to be self-compatible.

> PL2 <- pglmm(PL ~ PrePollinationCostsExclFruitSet + Self.compatibility + (1|GenusSpecies__), data = PollenLim, family = "gaussian", cov_ranef = list(GenusSpecies = tree.a$scenario.1), REML = TRUE, verbose = TRUE, s2.init = .1)

> summary(PL2)

Linear mixed model fit by restricted maximum likelihood

Call:PL ~ PrePollinationCostsExclFruitSet + Self.compatibility

logLik AIC BIC

-32.02 78.03 82.01

Random effects:

Variance Std.Dev

1|GenusSpecies 0.007927 0.08903

residual 0.317070 0.56309

Fixed effects:

Value Std.Error Zscore Pvalue

(Intercept) 0.19949 0.21757 0.9169 0.35920

Pre-pollination costs -0.23003 0.82076 -0.2803 0.77927

Breeding system:

Self-compatibility 0.39081 0.20149 1.9396 0.05243 .

Self-compatibility unknown 0.73182 0.48422 1.5113 0.13070

**S4 Table. Linear mixed model examining the influence of pre-pollination costs of seed production, including the effects of fruit set, on pollen limitation observed in nature (non-phylogenetic analysis).** The baseline condition is for plants to be self-compatible.

> Limitation3 <- lmer(PL ~ PrePollinationCostsInclFruitSet + Self.compatibility + (1|GenusSpecies), data = PollenLimCC)

> summary(Limitation3)

Linear mixed model fit by REML ['lmerMod']

Formula: PL ~ PrePollinationCostsInclFruitSet + Self.compatibility + (1 |GenusSpecies)

Data: PollenLimCC

REML criterion at convergence: 70

Scaled residuals:

Min 1Q Median 3Q Max

-1.5689 -0.4686 -0.1307 0.1976 4.1698

Random effects:

Groups Name Variance Std.Dev.

GenusSpecies (Intercept) 0.0000 0.0000

Residual 0.3275 0.5723

Number of obs: 41, groups: GenusSpecies, 17

Fixed effects:

Estimate Std. Error t value

(Intercept) 0.1043 0.1996 0.523

PrePollinationCostsInclFruitSet 0.2310 0.6776 0.341

Self.compatibilitySI 0.3559 0.1978 1.800

Self.compatibilityunknown 0.5447 0.5115 1.065

Correlation of Fixed Effects:

(Intr) PPCIFS Slf.SI

PrPllntCIFS -0.780

Slf.cmptbSI -0.107 -0.369

Slf.cmptblt 0.285 -0.561 0.361

optimizer (nloptwrap) convergence code: 0 (OK)

boundary (singular) fit: see help('isSingular')

> Limitation3.05 <- lmer(PL ~ Self.compatibility + (1|GenusSpecies), data = PollenLimCC)

> anova(Limitation3, Limitation3.05)

refitting model(s) with ML (instead of REML)

Data: PollenLimCC

Models:

Limitation3.05: PL ~ Self.compatibility + (1 | GenusSpecies)

Limitation3: PL ~ PrePollinationCostsInclFruitSet + Self.compatibility + (1 | GenusSpecies)

npar AIC BIC logLik deviance Chisq Df Pr(>Chisq)

Limitation3.05 5 76.509 85.077 -33.254 66.509

Limitation3 6 78.380 88.662 -33.190 66.380 0.1286 1 0.7199

**S5 Table. Linear mixed model examining the influence of pre-pollination costs of seed production, excluding the effects of fruit set, on pollen limitation observed in nature (non-phylogenetic analysis).** The baseline condition is for plants to be self-compatible.

> Limitation4 <- lmer(PL ~ PrePollinationCostsExclFruitSet + Self.compatibility + (1|GenusSpecies), data = PollenLimCC)

> summary(Limitation4)

Linear mixed model fit by REML ['lmerMod']

Formula: PL ~ PrePollinationCostsExclFruitSet + Self.compatibility + (1 |GenusSpecies)

Data: PollenLimCC

REML criterion at convergence: 69.7

Scaled residuals:

Min 1Q Median 3Q Max

-1.6552 -0.5261 -0.1241 0.1554 4.0912

Random effects:

Groups Name Variance Std.Dev.

GenusSpecies (Intercept) 0.0000 0.0000

Residual 0.3271 0.5719

Number of obs: 41, groups: GenusSpecies, 17

Fixed effects:

Estimate Std. Error t value

(Intercept) 0.2190 0.1976 1.109

PrePollinationCostsExclFruitSet -0.3132 0.7781 -0.402

Self.compatibilitySI 0.3985 0.1889 2.109

Self.compatibilityunknown 0.7190 0.4640 1.550

Correlation of Fixed Effects:

(Intr) PPCEFS Slf.SI

PrPllntCEFS -0.775

Slf.cmptbSI -0.236 -0.233

Slf.cmptblt 0.148 -0.410 0.273

optimizer (nloptwrap) convergence code: 0 (OK)

> Limitation4.05 <- lmer(PL ~ Self.compatibility + (1|GenusSpecies), data = PollenLimCC)

> anova(Limitation4, Limitation4.05)

Data: PollenLimCC

Models:

Limitation4.05: PL ~ Self.compatibility + (1 | GenusSpecies)

Limitation4: PL ~ PrePollinationCostsExclFruitSet + Self.compatibility + (1 | GenusSpecies)

npar AIC BIC logLik deviance Chisq Df Pr(>Chisq)

Limitation4.05 5 76.509 85.077 -33.254 66.509

Limitation4 6 78.330 88.611 -33.165 66.330 0.1791 1 0.6721
